# Supplementary material for: Machine Learning Accelerates Crystallization for Structure Determination
Source: Angew Chem Int Ed Engl. 2026 May 4;65(25):e1218503. doi: 10.1002/anie.1218503 (PMC13266958; doi:10.1002/anie.1218503)

checkCIF/PLATON report

Structure factors have been supplied for datablock(s) lcz011

THIS REPORT IS FOR GUIDANCE ONLY. IF USED AS PART OF A REVIEW PROCEDURE FOR PUBLICATION, IT SHOULD NOT REPLACE THE EXPERTISE OF AN EXPERIENCED CRYSTALLOGRAPHIC REFEREE.

No syntax errors found. CIF dictionary Interpreting this report

Datablock: lcz011

Bond precision:	C-C = 0.0068 A		Wavelength=1.54184
Cell:	a=8.2154(1)	b=16.7179(1)	c=24.4730(2)
	alpha=90	beta=97.001(1)	gamma=90
Temperature:	100 K		

	Calculated	Reported
Volume	3336.16(5)	3336.16(5)
Space group	P 21/n	P 1 21/n 1
Hall group	-P 2yn	-P 2yn
Moiety formula	C25 H15 Ag3 F18 N6 O4	C15 H3 Ag3 F18 N6, C10 H12 O4
Sum formula	C25 H15 Ag3 F18 N6 O4	C25 H15 Ag3 F18 N6 O4
Mr	1129.04	1129.04
Dx,g cm-3	2.248	2.248
Z	4	4
Mu (mm-1)	15.356	15.356
F000	2168.0	2168.0
F000’	2178.61	
h,k,lmax	10,21,31	10,21,30
Nref	7176	6887
Tmin,Tmax	0.140,0.100	0.570,1.000
Tmin’	0.039	

Correction method= # Reported T Limits: Tmin=0.570 Tmax=1.000
AbsCorr = MULTI-SCAN

Data completeness= 0.960 Theta(max)= 78.433

R(reflections)= 0.0362(6388) wR2(reflections)= 0.0898(6887)

S = 1.043 Npar= 509

The following ALERTS were generated. Each ALERT has the format
test-name_ALERT_alert-type_alert-level.
Click on the hyperlinks for more details of the test.

Alert level C

```
PLAT042_ALERT_1_C Calc. and Reported MoietyFormula Strings  Differ     Please Check
              Calc: C25 H15 Ag3 F18 N6 O4
              Rep.: C15 H3 Ag3 F18 N6, C10 H12 O4
PLAT213_ALERT_2_C Atom F1               has ADP max/min Ratio .....        3.9 prolat
PLAT213_ALERT_2_C Atom F01A             has ADP max/min Ratio .....        3.3 prolat
PLAT220_ALERT_2_C NonSolvent   Resd 1  F   Ueq(max)/Ueq(min) Range         4.7 Ratio
PLAT906_ALERT_3_C Large K Value in the Analysis of Variance ......       2.796 Check
PLAT911_ALERT_3_C Missing FCF Refl Between Thmin & STh/L=    0.600          13 Report
               -1  0  1,  -2  2  1,   0  1  2,  -9  0  9,  -8 10 11,  -9  1 12,
               -7  0 15,  -5  5 15,  -6  0 16,  -6  0 22,  -7  0 23,   1  4 28,
                0  5 28,
```

Alert level G

```
PLAT007_ALERT_5_G Number of Unrefined Donor-H Atoms .............          1 Report
              H00F
PLAT083_ALERT_2_G SHELXL Second Parameter in WGHT  Unusually Large      15.97 Why ?
PLAT142_ALERT_4_G s.u. on b - Axis Small or Missing .............    0.00010 Ang.
PLAT143_ALERT_4_G s.u. on c - Axis Small or Missing .............    0.00020 Ang.
PLAT242_ALERT_2_G Low    'MainMol' Ueq as Compared to Neighbors of       C01B Check
PLAT242_ALERT_2_G Low    'MainMol' Ueq as Compared to Neighbors of       C01E Check
PLAT242_ALERT_2_G Low    'MainMol' Ueq as Compared to Neighbors of       C01F Check
PLAT242_ALERT_2_G Low    'MainMol' Ueq as Compared to Neighbors of       C01I Check
PLAT242_ALERT_2_G Low    'MainMol' Ueq as Compared to Neighbors of       C01J Check
PLAT380_ALERT_4_G Incorrectly? Oriented X(sp2)-Methyl Moiety .....       C01K Check
PLAT434_ALERT_2_G Short Inter HL..HL Contact F007     ..F008     .       2.82 Ang.
                                              2-x,1-y,1-z  =      3_766 Check
PLAT720_ALERT_4_G Number of Unusual/Non-Standard Labels ..........          68 Note
              Ag01    Ag02    Ag03    F004    F005    F006    F007    F008
              F009    F00A    F00B    F00C    F00E    O00F    H00F    O00G
              F00H    O00I    N00J    N00K    F00L    F00M    N00N    N00O
              N00P    N00Q    O00R    F00S    C00T    C00U    H00U    C00V
              C00W    C00X    H00X    C00Y    C00Z    C010    C011    C012
              C014    H014    C015    C016    C017    C018    C019    H019
              F01A    C01B    C01C    H01A    H01B    H01C    C01D    H01D
              C01E    C01F    C01H    H01E    H01F    H01G    C01I    C01J
              C01K    H01H    H01I    H01J
PLAT883_ALERT_1_G Absent Datum for _atom_sites_solution_primary ..      Please Do !
PLAT912_ALERT_4_G Missing # of FCF Reflections Above STh/L=  0.600         274 Note
PLAT913_ALERT_3_G Missing # of Very Strong Reflections in FCF ....           1 Note
               -2  2  1,
PLAT941_ALERT_3_G Average HKL Measurement Multiplicity ..........         3.1 Low
```

```
PLAT969_ALERT_5_G The 'Henn et al.' R-Factor-gap value ..........      3.295 Note
              Predicted wR2: Based on SigI**2  2.72 or SHELX Weight  8.61
PLAT978_ALERT_2_G Number C-C Bonds with Positive Residual Density.         0 Info
```

```
   0 ALERT level A = Most likely a serious problem - resolve or explain
   0 ALERT level B = A potentially serious problem, consider carefully
   6 ALERT level C = Check. Ensure it is not caused by an omission or oversight
  18 ALERT level G = General information/check it is not something unexpected

   2 ALERT type 1 CIF construction/syntax error, inconsistent or missing data
  11 ALERT type 2 Indicator that the structure model may be wrong or deficient
   4 ALERT type 3 Indicator that the structure quality may be low
   5 ALERT type 4 Improvement, methodology, query or suggestion
   2 ALERT type 5 Informative message, check
```

Validation response form

Please find below a validation response form (VRF) that can be filled in and pasted into your CIF.

```
# start Validation Reply Form
_vrf_PLAT042_lcz011
;
PROBLEM: Calc. and Reported MoietyFormula Strings  Differ     Please Check
RESPONSE: ...
;
_vrf_PLAT213_lcz011
;
PROBLEM: Atom F1              has ADP max/min Ratio .....        3.9 prolat
RESPONSE: ...
;
_vrf_PLAT220_lcz011
;
PROBLEM: NonSolvent   Resd 1  F   Ueq(max)/Ueq(min) Range        4.7 Ratio
RESPONSE: ...
;
_vrf_PLAT906_lcz011
;
PROBLEM: Large K Value in the Analysis of Variance ......      2.796 Check
RESPONSE: ...
;
_vrf_PLAT911_lcz011
;
PROBLEM: Missing FCF Refl Between Thmin & STh/L=    0.600         13 Report
RESPONSE: ...
;
# end Validation Reply Form
```

It is advisable to attempt to resolve as many as possible of the alerts in all categories. Often the minor alerts point to easily fixed oversights, errors and omissions in your CIF or refinement strategy, so attention to these fine details can be worthwhile. It is up to the individual to critically assess their own results and, if necessary, seek expert advice.

PLATON version of 17/09/2025; check.def file version of 17/09/2025

duplicate check

No duplication found

Datablock lcz011 - ellipsoid plot

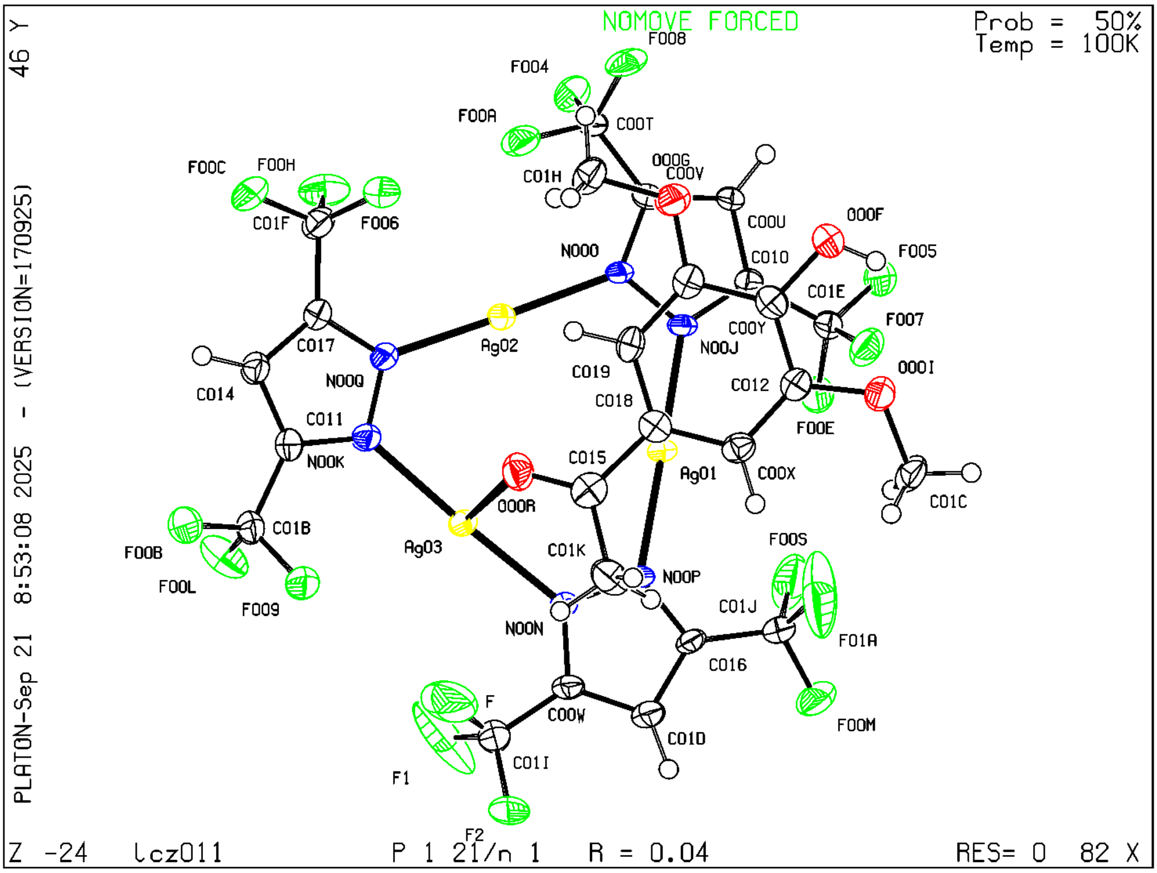

Supplement: Supplementary file 2 — Supporting File 2: anie72462‐sup‐0002‐DataFile.zip. [file ANIE-65-e1218503-s002.zip › supplementary_materials_crystallographic_data submit.zip!/cif_and_checkcif(1-114)/Ag3Pz3_11/Ag3Pz3_11_checkcif.pdf]
